# Role of gold nanoparticles in advanced biomedical applications

**DOI:** 10.1039/d0na00472c

**Published:** 2020-07-16

**Authors:** Suneev Anil Bansal, Vanish Kumar, Javad Karimi, Amrinder Pal Singh, Suresh Kumar

**Affiliations:** Department of Mechanical Engineering, University Institute of Engineering and Technology (UIET), Panjab University Chandigarh India 160014 suneev@gmail.com; Department of Mechanical Engineering, MAIT, Maharaja Agrasen University HP India 174103; National Agri-Food Biotechnology Institute (NABI) S. A. S. Nagar Punjab 140306 India; Department of Biology, Faculty of Sciences, Shiraz University Shiraz 71454 Iran; Department of Applied Science, University Institute of Engineering and Technology (UIET), Panjab University Chandigarh India 160014 skphysicsnano@gmail.com

## Abstract

Gold nanoparticles (GNPs) have generated keen interest among researchers in recent years due to their excellent physicochemical properties. In general, GNPs are biocompatible, amenable to desired functionalization, non-corroding, and exhibit size and shape dependent optical and electronic properties. These excellent properties of GNPs exhibit their tremendous potential for use in diverse biomedical applications. Herein, we have evaluated the recent advancements of GNPs to highlight their exceptional potential in the biomedical field. Special focus has been given to emerging biomedical applications including bio-imaging, site specific drug/gene delivery, nano-sensing, diagnostics, photon induced therapeutics, and theranostics. We have also elaborated on the basics, presented a historical preview, and discussed the synthesis strategies, functionalization methods, stabilization techniques, and key properties of GNPs. Lastly, we have concluded this article with key findings and unaddressed challenges. Overall, this review is a complete package to understand the importance and achievements of GNPs in the biomedical field.

## Abstract 2

Gold nanoparticles (GNPs) have generated keen interest among researchers in recent years due to their excellent physicochemical properties.

## Graphical abstract

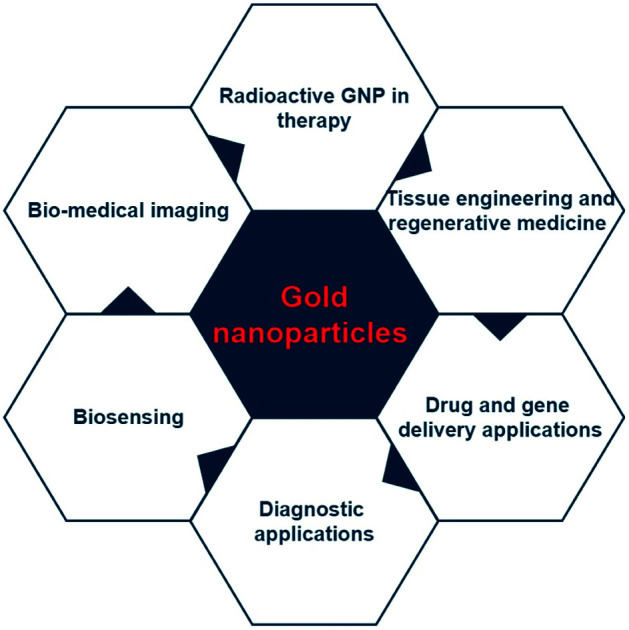

## 1. Introduction

The advent of nanotechnology has increased our capability to engineer the physicochemical properties of materials at the nano-scale to enable their use in various biomedical applications.^1–3^ Out of all the nanomaterials, gold nanoparticles (GNPs) and silver nanoparticles are the most explored nanostructures for biomedical applications.^4,5^ Among the two aforementioned metal nanostructures, the scope of nano-silver (in biomedical applications) is limited in comparison to GNPs due to its higher cytotoxicity and low colloidal stability.^6,7^ The physical and chemical properties of GNPs are largely different from those of bulk gold.^8^ So far, the unique properties of GNPs have been exploited for many advanced biomedical applications (*e.g.*, *in vitro*/*in vivo* use in bio-imaging, gene delivery, contrast enhancement of X-ray computed tomography, targeted drug delivery, diagnostics, plasmonic bio-sensing, colorimetric sensing, tissue engineering, photo-induced therapy, and cancer therapy).^9,10^ Even though the medicinal applications of gold have been known to us since ancient human civilizations, recent advances in the field of nanotechnology have further facilitated its use in the biomedical field (*e.g.*, biomedical imaging, targeted (site specific) drug delivery, and photo-induced therapeutic applications).^11,12^

**Fig. 1:**
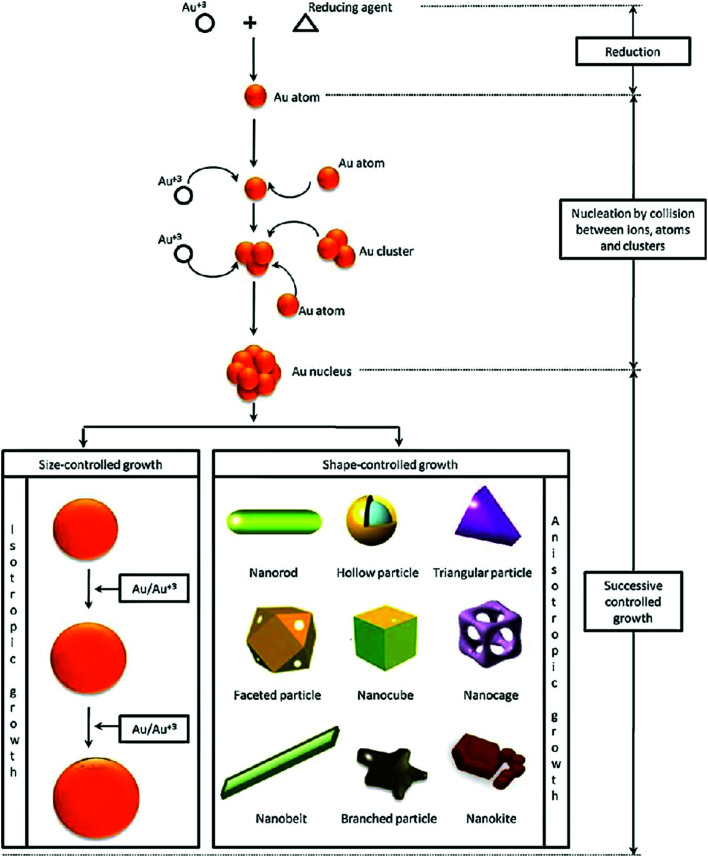
Synthesis procedure of gold nanoparticles with different shapes, using a chemical bottom-up approach.^17^ This figure has been adapted/reproduced from ref. 17 with permission from Elsevier, copyright 2011.

Generally, the physical and electronic properties of GNPs (and other gold nanostructures such as nanostars, nanorods, nanocages, nanoshells, and nanoprisms) are largely dependent upon their size and shape.^13–17^ Images of different types of gold nanostructures are shown in Fig. 1. For instance, the interaction of GNPs with electromagnetic radiation is surprisingly different from that of their bulk counterparts.^18^

Likewise, diversity in the color of GNPs can be seen on the basis of their size and shape, while bulk gold exhibits only a bright yellow color.^19,20^ The variations in the band gap with size have been majorly responsible for such variations in the properties of gold in the nano form. Usually, the band gap of nanoparticles increases with decreasing particle/cluster size.^21^ When the size of nanoscale gold approaches the Fermi wavelength (∼0.5 nm), the energy levels become discrete enough to affect their electronic properties.^22,23^ Most of the biomedical engineering applications of gold nanoparticles are based on their unique size dependent optical properties.^22,24^ The scope of the GNPs in biomedical applications is vast enough to cover molecular level cellular imaging to the treatment of cancer-like diseases.^22,25–30^ Likewise, theranostic applications (bio-imaging + therapy) of gold based nanostructures are attracting interest in many clinical applications.^31–33^ The potential of GNPs in biomedical applications is promising but there are still many issues (*e.g.*, immunogenicity and cytotoxicity of the GNPs) that need to be addressed in order to realize their full potential.^34–36^ Nonetheless, use of chemical/biomolecule functionalized GNPs is an effective way to overcome the above-mentioned issues.^37–39^

Owing to their exciting and unique physicochemical properties, GNPs are sure to contribute profoundly to the field of biomedical engineering. Specifically, functionalized GNPs are found to be more beneficial for biomedical applications due to their excellent bio-compatibility and colloidal stability.^6,40^ So far, a number of GNP based advanced modalities have been developed for healthcare applications (for diagnostics as well as therapeutics).^41–43^ Considering the extraordinary potential of GNPs in the biomedical field, we designed this review to highlight the latest achievements of GNPs in the biomedical arena. Special focus has been given to emerging biomedical applications including bio-imaging, site specific drug/gene delivery, nano-sensing, diagnostics, and photon induced therapeutics. We have also summarized the basics of gold metal, presented a historical overview of GNPs, and discussed GNP synthesis strategies. Lastly, we have concluded this review with key findings and unaddressed challenges. A few reviews have already been published to highlight the potential of GNPs in the biomedical field.^4,44–49^ However, no recent review explaining the biomedical advancements of GNPs considering historical views, synthesis and modification strategies, and key biomedical achievement is available to spotlight the current advancements in this area. Herein, we have provided up-to-date information on the progress of GNPs for biomedical applications.

## 2. Gold metal and nanogold: early historical uses

Gold has a face centered cubic crystal structure and the properties of gold are very different from those of other fellow elements of the group.^50,51^ Generally, gold (a noble metal) is considered as a symbol of immortality.^52^ Gold is also considered to be non-toxic to humans and allowed as a healthy food additive.^25^ Interestingly, gold when reduced to a nano-scale size changes its physicochemical properties dramatically.^22,53^ The most famous masterpiece example displaying a color change on reduction of gold and silver particle size is the Lycurgus cup (displayed at the British Museum, London).^54,55^ By fine-scale size tuning of gold, the surface plasmon resonance (SPR) absorption can be altered from the visible to the NIR region of the electromagnetic spectrum.^56^ In the 1850s, colloidal gold was first prepared and named divided gold metal (mentioned is his diary) by Michael Faraday.^55,57^ Furthermore, Mie explained that the brilliant color of Faraday's gold solution is due to absorption and scattering of light by the nano-sized particles.^22,57^ This unique colorimetric property of GNPs has been used in biological systems, *e.g.*, in cellular uptake investigations.^58^ It was revealed later that GNPs exhibit size and shape dependent optical and electronic properties due to the SPR effect.^59^

**Fig. 2:**
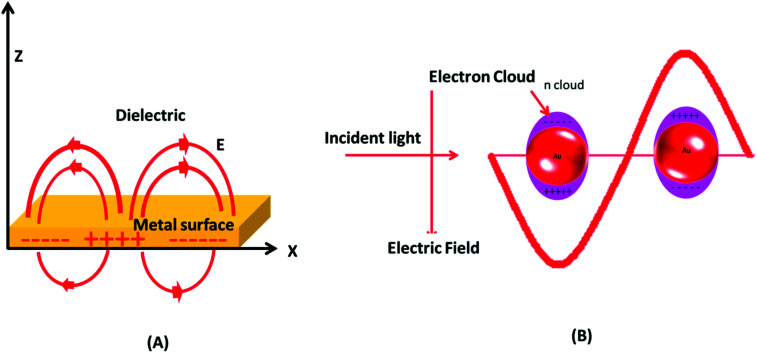
Surface plasmon resonance in gold nanoparticles. (A) Schematic of a propagating surface plasmon polariton and (B) localized surface plasmon resonance.

The visible light absorption spectrum of gold nanoparticles gives a strong SPR absorption band around 520 nm. The absorption band is sensitive to the size of nanoparticles and surface modifications (*e.g.*, with bio-molecules). These optical properties of GNPs have also been successfully validated and explained with several theoretical studies.^60^ It was postulated that the surface electrons of gold nanostructures are excited after interacting with electromagnetic radiation and start vibrating with the frequency of impinging radiation (Fig. 2). This phenomenon is termed as localized surface plasmon resonance (LSPR). Note that absorption and scattering of electromagnetic radiation occur as a result of interaction of light rays with nano-scale gold structures. The LSPR of GNPs is found to be crucial for several advanced innovations in the biomedical field. The optically sensitive properties of GNPs have also been utilized in surface enhanced Raman scattering (SERS), especially for sensing applications.^61^ It is suspected that the plasmonic nanoparticles enhance the local electric field in their close proximity, which increases the magnitude of the Raman signal of the molecules lying in close proximity to the nanoparticles. This enhancement in Ramam signals enhances the sensing capability of such systems.^62^

The properties of gold metal change significantly as the size is reduced to the nano-scale (1–100 nm); this range results in much more than just a decorative metal piece.^20,63^ In recent years, fine-scale GNPs have attracted tremendous interest from biomedical researchers owing to their useful and highly desirable physicochemical properties such as quantized charging and discharging, non-toxicity, high surface area to volume ratio, easy surface modification, photon induced heating, electromagnetic wave absorption and scattering, and optical and electronic properties.^22,25,53,64,65^ In the modern era, GNPs have been widely used in biomedical fields.^22,53,62^ The excellent characteristics of GNPs are sufficient enough to find applications in almost every field of biomedical diagnostics and therapeutics. Potential examples of GNP applications in biomedicals include but are not limited to dental implants, X-ray contrast enhancing agents in computed tomography, photoacoustic bio-imaging, electron microscopy, bio-sensing, photothermal/photodynamic therapy, targeted drug delivery, and ultrasensitive chemical/bio-sensing.^8,66–69^

In general, the high surface area to volume ratio is the most common property of nanoparticles that make them more useful than their bulk counterparts.^70^ Biomedical engineering is an integral part of today's health care industry and plasmonic gold nanoparticles or gold coatings are extensively used in this field. GNPs efficiently absorb impinging photons and transduce the photon energy to thermal energy, which can be utilized in thermal killing of cancerous cells.^71^ By interfacing suitable bio-molecules to the surface of GNPs, specific malignant cells can be targeted for removal without harming the healthy cells.^72^ It has been investigated that for the destruction of cancerous cells, rod shaped gold nanoparticles are more effective in comparison to spherical shaped gold nanostructures.^72^

**Fig. 3:**
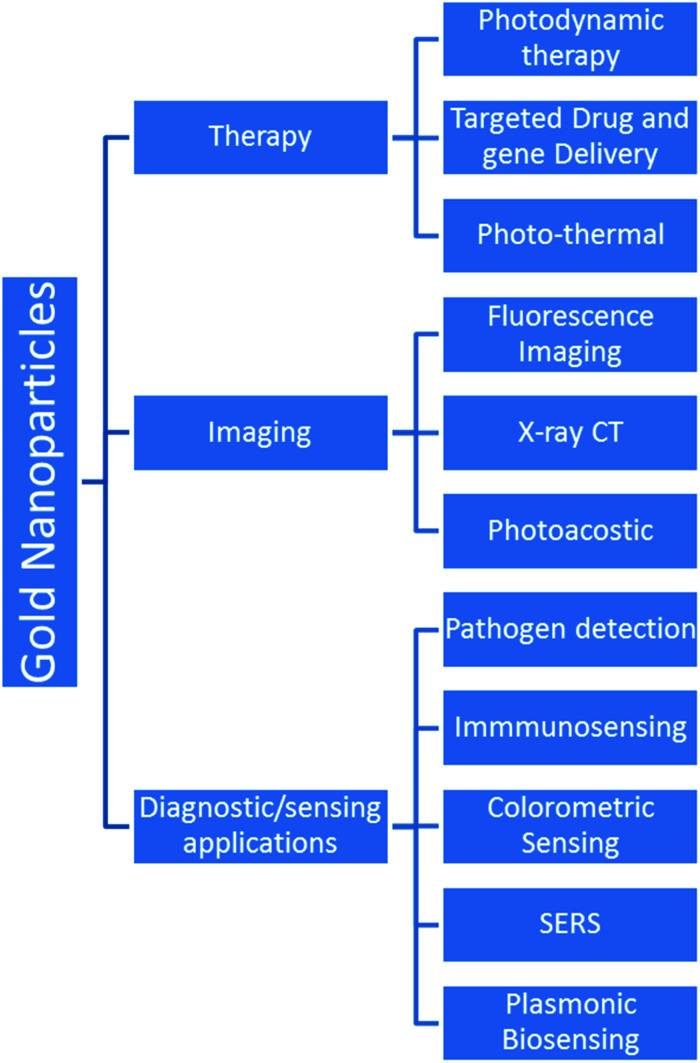
Various advanced applications of gold nanoparticles.

The GNPs also offer the benefit of easy functionalization, which allows the facile attachment of the desired molecule on their surface.^73,74^ Interestingly, the attachment of specific molecules (*e.g.*, antibodies) on the GNP surface can be used to deliver the drug molecules/biological moieties to desired locations in the human body. Moreover, modified/unmodified GNPs have also been utilized successfully in bio-imaging applications.^41^ GNP based major potential applications in biomedical engineering are shown in Fig. 3.

## 3. Properties of gold nanoparticles

The unique and extraordinary properties of GNPs are the basis of their large scale biomedical applications. GNPs exhibit diverse physical, chemical, and biological properties.

### 3.1. Physical and chemical properties

GNPs are well known for their excellent physical and chemical properties.^75–79^ In the biomedical field, the optical properties of GNPs are among the most explored physical properties.

Excitingly, the bulk form of gold is yellow in color, while finely divided GNPs exhibit a size tunable color ranging from violet to wine red.^20,81^ These variations in color in GNPs can be correlated with photophysical phenomena, namely SPR and/or LSPR.^82^ SPR arises from the surface propagation of plasmon waves, also known as surface plasmon polaritons, across a thin metal surface. LSPR is plasmon oscillation in a metal nanocrystal.^25,83^ Both LSPR and SPR can be used for efficient biological sensing applications. The color change from bright yellow (bulk) to other size-dependent brilliant colors is attributed to LSPR. The reduction of particle size below the wavelength (*λ*) of incident electromagnetic radiation gives rise to a plasmonic phenomenon. The interaction between electromagnetic rays and metal nanoparticles can be explained by Mie's theory.^84–86^ In brief, Mie postulated that the color in colloidal gold nanoparticles was due to absorption and scattering of light by nano-sized particles.^22,57,86^ Furthermore, R. Gans extended Mie's theory by including non-spheroidal shaped particles. R. Gans extended Mie's theory to calculate the values of extinction and scattering coefficients for both prolate and oblate ellipsoids.^85,87,88^

**Fig. 4:**
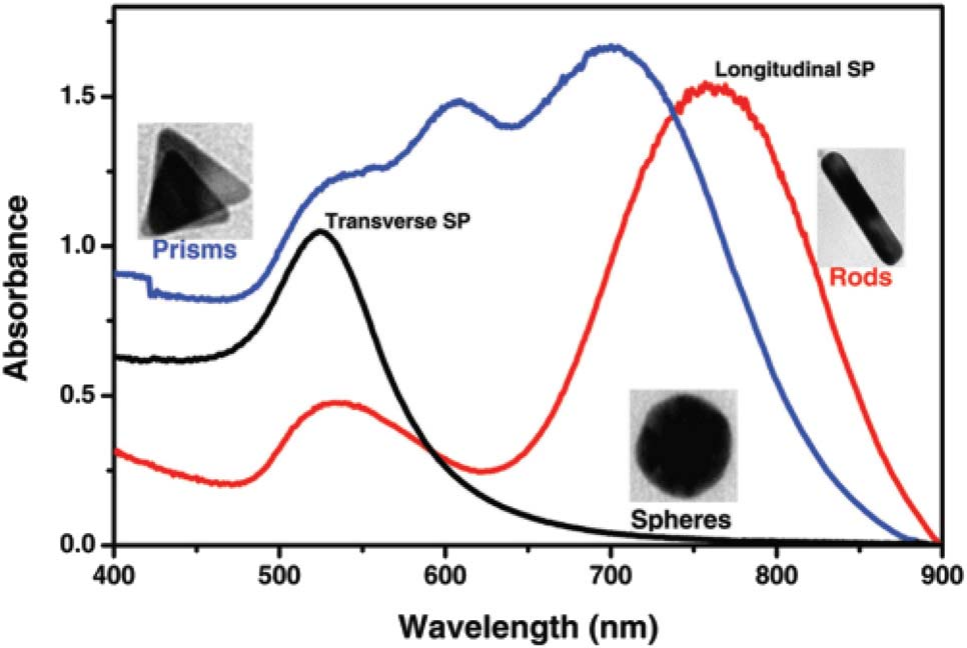
Localized surface plasmon resonance absorption band for gold spherical nanoparticles (black line), nanorods (red line) and prisms (blue line).^80^ This figure has been adapted/reproduced from ref. 80 with permission from RSC, copyright 2015.

As shown in eqn (1), the strength of the LSPR signal and *λ*_max_ of metal nanoparticles depend upon the size of the particles, metal used (the signal is strong in noble metals like gold and silver), dielectric constant of the metal/medium, extinction coefficient, and density of electrons (surface charge).^81^1
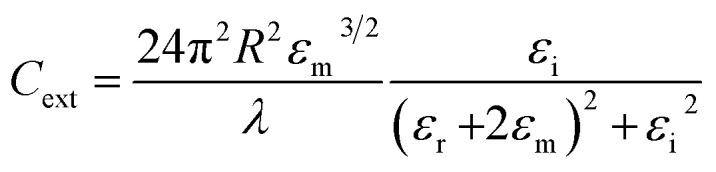
where *C*_ext_ is the extinction cross-section, *R* is the radius of the nanoparticle, *ε*_m_ is the dielectric constant of the medium in the vicinity of the metal nanoparticle, *λ* is the wavelength of incident electromagnetic radiation, *ε*_r_ is the real part of the metal dielectric constant, and *ε*_i_ is the imaginary part of the metal dielectric constant. In any metal, SPR occurs when the *ε*_r_ = −2*ε*_m_ condition is satisfied.^81^ On the basis of eqn (1), for a given metal nanoparticle, *ε*_r_ decides SPR *λ*_max_ and *ε*_i_ decides the width of the LSPR band.^81^ Moreover, LSPR can be tuned by changing the shape and size of the nanoparticle. For example, the nanoparticles of spherical GNPs appear at around 520 nm (UV-vis spectrum), while in the case of gold nanorods, two absorption bands appear due to transverse LSPR (at lower wavelength) and longitudinal SPR (at higher wavelength) (Fig. 4).^80^ Likewise, as evident from Fig. 4, prism-like structures reflect a higher number of LSPR bands.^80^

Both scattering and absorption occur simultaneously during electromagnetic radiation interaction with gold nanoparticles represented by a single term known as extinction.^89^ Extinction, scattering and absorption cross-sections of GNPs can be easily evaluated by carrying out theoretical calculations. Experimentally, the UV-Vis-NIR spectrum of GNPs gives information about the extinction cross-section. Table 1 shows the LSPR of GNPs with various sizes and shapes.^90,91^ These LSPR values are very crucial while designing GNP related experiments in biomedical fields.

**Table 1:** LSPR and axial surface plasmon resonance (ASPR) of gold based nanostructures

Type of nanocrystal	Dimension (nm)	Peak SPR wavelength (nm)	LSPR molar ext (M^−1^ cm^−1^)	ASPR molar ext (M^−1^ cm^−1^)	Ref.
Gold nanoparticles	5–100	LSPR: 515–572	1.10 × 10^7^ to 1.57 × 10^11^	—	90
Gold nanorods	Axial diameter: 10	ASPR: 512	8.99 × 10^8^ to 1.02 × 10^9^	2.25 × 10^8^ to 2.55 × 10^8^	91
	Longitudinal length: 29–41	LSPR: 700–808			
	Axial diameter: 25	ASPR: 530	9.15 × 10^8^ to 4.58 × 10^9^	5.72 × 10^8^ to 2.29 × 10^9^	91
	Longitudinal length: 34–73	LSPR: 550–700			
Gold clusters	<2	—	—	—	22
Nanoshells	10–400	520–900	—	—	22
Gold nanocages and boxes	20–200	400–1200	—	—	92
Branched gold nanostructures	45–300	550–800			22

### 3.2. Biological properties of gold nanoparticles: toxicity aspect

GNPs as such are non-toxic but the GNP solution can be toxic due to the stabilizing capping agents and chemical remnants used for synthesizing GNPs.^93,94^ For example, CTAB used in stabilizing gold nanorods causes toxicity even at nano-molar concentration.^95^ Nevertheless, toxicity can be reduced by the replacement of a toxic capping agent by a suitable biocompatible capping agent or by modifying CTAB to prevent its dissolution.^96^ In a report on the cytotoxicity of citrate coated GNPs, it was observed that these particles are non-cytotoxic at low concentration. Toxicity also depends on the size and concentration of GNPs.^97^ The investigation of interactions of GNPs with the contents of biological medium is important from the perspective of their biomedical engineering applications.^96^ GNPs whose size is <2 nm are found to cause oxidative damage to the mitochondrial structure and lead to the destruction of biological cells.^96^

Cytotoxicity study of a material, which is to be used in the biomedical field, is highly important. Different size and shape GNPs have been used by many researchers in diverse biomedical applications.^92,98,99^ As discussed in other sections of this review, radioactive and simple gold nanostructures have been used in many advanced biomedical applications.^100^

Alkilany and co-workers studied the toxicity of colloidal GNPs.^58^ It was observed that the dispersing medium can also cause cytotoxicity and therefore the cytotoxicity of GNPs and the dispersion medium should be compared separately. Toxicity was found to be size and shape dependent. For example, 1.4 nm sized GNPs were found to be toxic but 15 nm GNPs were not.^102^ Various experimental reports suggested that the toxicity is dose dependent and the safety concentration limit is 1012 particles per mL.^103^ The nature of interaction between GNPs and bio-systems depends upon the synthesis procedure, size/shape, charge on the surface and type of surface coating.^104^ In many reports GNPs have been considered as non-toxic carriers for various drugs, nucleic acids, and gene delivery applications.^105^ GNPs can also infiltrate through human skin and cell membranes. Interestingly, polyethylene glycol (PEG) and ionic coatings (zwitter-ionic) on GNPs improved their biocompatibility.^106^ Explaining the toxic effect of GNPs on a biological system is very difficult and still more research is required in this research area.

The synthesis method of GNPs also affects their toxicity. For example, GNPs made from plant extracts, algae, and bacteria are less or non-toxic and considered suitable for biomedical applications.^107^ In certain cases, cell killing capabilities (for cancerous cells) have been imparted into GNPs by functionalization with suitable molecules. For example, Kong *et al.* modified GNPs with cysteamine and thio-glucose.^101^ The cysteamine modified GNPs were tethered to human breast adenocarcinoma line MCF-7 cell membrane while Glu-GNPs easily crossed the cell membrane and spread throughout the cytoplasm.^101^ Functionalized GNPs showed almost no toxic effect even for hours under normal body conditions and environment. These entrapped nanoparticles were then irradiated with X-rays and gamma rays to effectively kill cancerous cells.

In another report, researchers modified 10 nm GNPs with glutathione to make them toxic to prohibit cell proliferation at high concentration.^63^ It has been observed that compared to 1.4 nm GNPs, 15 nm GNPs are non-toxic even at higher concentration (∼60 fold lower in comparison to 1.4 nm GNPs). The safe concentration limit was even more enhanced (∼100 fold) when GNPs were modified with thiomalate.^108^ Lee *et al.* have also studied the size and shape dependent cytotoxicity of GNPs prepared by using green tea extract.^109^ They used nanostars, nanorods, and nanospheres and studied cytotoxicity in cancerous cells. Uptake of nanostructures by cells follows the order nanospheres > nanorods > nanostars. 3-(4,5-Dimethylthiazol-2-yl)-2,5-diphenyltetrazolium bromide (MTT) assay was performed to evaluate the cytotoxicity of these gold nanostructures. Cytotoxicity followed the order nanorods > nanostars > nanospheres. It can be concluded from this research that spherical nanoparticles are most suitable for biomedical applications.^109^ In another report, *in vitro* size dependent cytotoxicity was evaluated.^110^ Citrate ion coated GNPs of sizes 3, 8 and 30 nm were found to be cytotoxic, while 5, 10, 17 and 45 nm sized GNPs were found to be non-toxic at higher concentrations.^110^ In another work, poly(methacrylic acid) modified 4 nm GNPs were exposed to biological cells.^111^ It was observed that dose concentration higher than 50 nM reduces cell viability and cell proliferation.^111^ The type of functionalization is also important in deciding GNP toxicity. For instance, citrate capped GNPs aggregated more rapidly in comparison to starch and gum-arabic modified GNPs.^112^ Aggregation of GNPs considerably affects the cell viability.^112^

## 4. Synthesis and colloidal stability of gold nanoparticles

### 4.1. Synthesis of gold nanoparticles

**Fig. 5:**
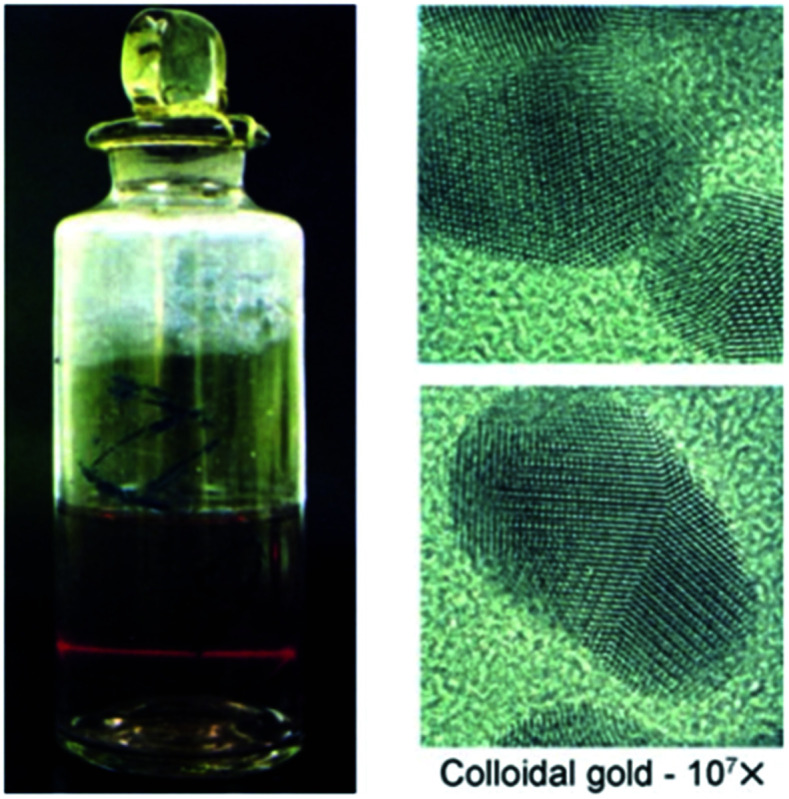
Faraday's colloidal gold showing the Tyndall effect and transmission electron micrograph of the GNPs.^116^ This figure has been adapted/reproduced from ref. 116 with permission from John Wiley and Sons, copyright 2007.

Bottom-up chemical synthesis of GNPs is the most common and convenient approach used by researchers.^113,114^ Generally, precise control over the size, shape, and dispersion is a decisive factor that defines the effectiveness of a particular synthesis method. Faraday's method is possibly considered as the first technique used to produce GNPs in the form of a colloid. In Faraday's approach NaAuCl_4_ was reduced with phosphorus/carbon disulfide solution to prepare a ruby color solution of gold.^115^ The ruby color of the resultant solution was suspected due to the formation of fine GNPs and their special interaction with the electromagnetic radiation. A digital image of Faraday's colloidal gold solution (preserved in the Faraday Museum) is shown in Fig. 5.

Later on, citrate capped GNPs were prepared by a hydrothermal reduction method.^117^ In this method, sodium citrate was used to reduce HAuCl_4_ (tetrachloroauric acid) under reflux conditions. Sodium citrate acts as a reducing agent as well as a capping agent for the GNPs. The above-mentioned synthesis approach is commonly known as the Turkevich method. Interestingly, citrate coating on the GNPs led to better dispersion of the nanoparticles.^117^ Likewise, many advancements in hydrothermal synthesis methods have been achieved for fabrication of GNPs.^118–120^ These approaches were also found effective to generate other gold nanostructures such as nanoplates and nanoshells.^14,22,62,121^

**Fig. 6:**
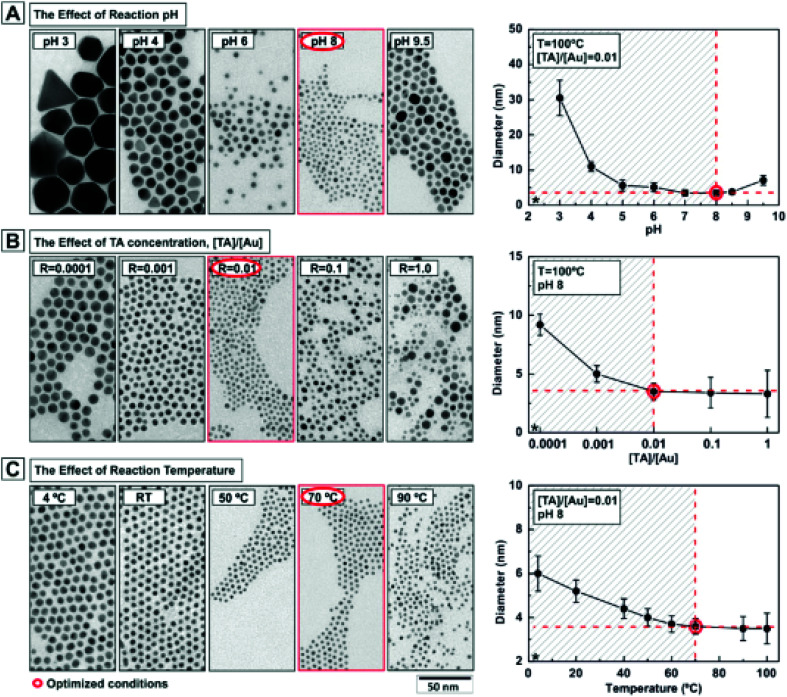
Effect of reaction parameters on the characteristics of gold nanoparticles. Effect of (A) pH, (B) tannic acid concentration and (C) temperature on the size of gold nanoparticles.^119^ This figure has been adapted/reproduced from ref. 119 with permission from the American Chemical Society, copyright 2016.

The use of small amounts of polyphenols, *e.g.*, tannic acid, along with sodium citrate is found favorable for obtaining fine (*e.g.*, 3.5 nm sized) particles of GNPs.^119^ Several factors, *e.g.*, solution pH, temperature of the solution, and concentration of reducing agent are crucial to obtain GNPs with the desired characteristics.^119^ The effect of pH values, tannic acid concentrations, and reaction temperature on the particle size of GNPs is shown in Fig. 6.

A further improvement in the Turkevich method for GNP synthesis was obtained by the addition of silver ions during the synthesis process.^122^ The mixing of HAuCl_4_ with a small amount of AgNO_3_ in aqueous solution led to the formation of a black colored solution. After incubation for a short time interval (∼5 min), the mixture was allowed to react with hot sodium citrate solution under vigorous stirring. The addition of silver ions led to the blocking of secondary nucleation growth to produce fine GNPs. This method is effective for the production of GNPs with sizes ranging from 10 to 36 nm.^53,122^

Likewise, chemical reduction was used for the synthesis of GNPs by employing HAuCl_4_·3H_2_O : HCl (in equal molar ratio) and NaBH_4_ : NaOH (in equal molar ratio).^123^ Note that HCl and NaOH were used to increase the stability of the solution for a longer time. The GNPs were produced by adding the gold salt/acid solution into water under continuous stirring conditions. The reaction was followed by the addition of acetone, hexane, and 1-dodecanethiol. After vigorous shaking the nanoparticles transfer to the hexane phase. Interestingly, byproducts of the synthesis reaction remain in the aqueous phase and therefore eliminate the post-synthesis washing procedure.^123^ In another chemical reduction approach, a two-step synthesis process was used to prepare GNPs.^20^ In the first step, sodium acrylate was used to reduce HAuCl_4_ to obtain 15 nm Au seeds. In the second step, bigger size nanoparticles were developed by reducing Au ions on Au seeds by adding acrylic acid to a Au seed/sodium acrylate mixture.^20^ This method was devised to provide better control over the size of GNPs.

Interestingly, a seed mediated multistep method can also be used to produce nanocrystals with different aspect ratios.^124^ In the first step, the growth solution was prepared by mixing HAuCl_4_, cetyl-tri-methyl ammonium bromide (CTAB), cyclohexane, and acetone. In the second step, gold seeds with very fine size were produced using conventional reducing agents, *e.g.*, sodium citrate and sodium borohydride. In the next step, different concentrations of seed solutions were added along with ascorbic acid and AgNO_3_ to the growth solution. Note that ascorbic acid acts as a reducing agent.^124,125^ The size and shape of the resulted GNP crystals depend upon the amount of seed, concentration of gold salt, and purity of the CTAB.^124,126^ In seed mediated synthesis, gold nanostructures with different shapes and sizes such as bipyramids, stars, cubes, and dodecahedrons can be prepared by varying the concentration of gold precursor, ascorbic acid, capping agent, and seed.^53,127^ Likewise, the photochemical process is also effective for the seed mediated synthesis of gold nanoparticles.^128^

The interfacial synthesis approach is another approach used successfully for the synthesis of GNPs, in which two phases (aqueous and organic phases) were used to prepare controlled dimensions of GNPs.^129^ In this two-phase synthesis method, tetraoctylammonium bromide (TOAB) was used to transfer AuCl_4_^−^ ions to the toluene layer from the aqueous phase. Upon addition of sodium borohydride (aqueous solution) in the presence of dodecanethiol, the color of the organic layer changes from yellow due to the formation of GNPs. The formation of nanoparticles occurs at the aqueous–organic interface. Note that dodecanethiol was used to cap nanoparticles, which was helpful in preventing their coagulation.^129^

Laser ablation and arc discharge are other good methods used to prepare GNPs.^130–132^ The laser-based GNP synthesis is simple and cost-effective. In this method, a bulk gold target immersed in liquid is ablated with a strong laser to obtain GNPs.^130^ The energy of the laser photon is transferred to the target material at the solid/liquid interface. Surfactants can also be used in aqueous solution during laser ablation to provide better control over the size of nanoparticles and dispersion in solution. It was observed that in the case of laser ablation, the size of the nanoparticles depends upon the size of surfactant molecules, laser fluence, and concentration of the surfactant.^133,134^

**Fig. 7:**
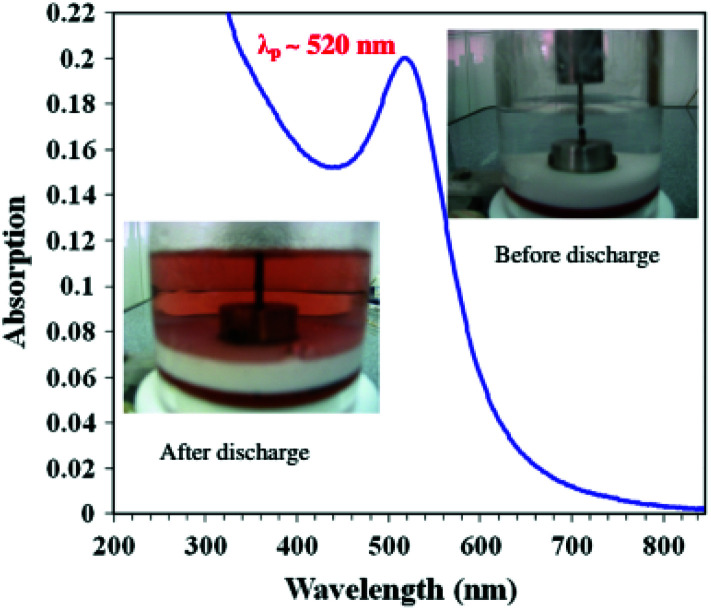
Arc discharge method setup for the production of GNPs.^131^ This figure has been adapted/reproduced from ref. 131 with permission from Springer Nature, copyright 2012.

Like the laser ablation method, the arc discharge method is also carried out in solution.^132^ In this, an arc discharge is applied between gold metal electrodes in aqueous and anhydrous ethanol medium to prepare GNPs. This method was further modified by reducing HAuCl_4_ by plasma discharge.^135^ In the arc discharge method, sodium citrate was also used as a stabilizing agent.^131,132^ The basic setup for the arc discharge method is shown in Fig. 7.^131^

**Fig. 8:**
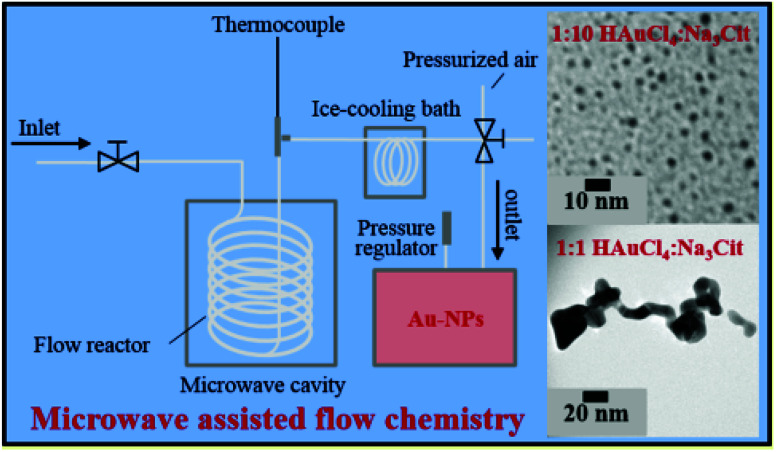
Experimental setup for microwave assisted synthesis of gold nanoparticles.^121^ This figure has been adapted/reproduced from ref. 121 with permission from the American Chemical Society, copyright 2016.

The use of microwaves is also found to be effective to generate GNPs.^121^ In microwave assisted GNP synthesis, microwave heating was used for the initiation/progression of reaction between HAuCl_4_ and sodium citrate in a flow reactor arrangement. The heated solution was then immediately cooled in an ice bath to stop the reaction.^121^ The size and shape of the obtained nanoparticles was reliant on the amount of reducing agent added. Moreover, the aspect ratio of the particles was found to depend on microwave power. The experimental arrangement of the synthesis device used for the microwave synthesis of GNPs is shown in Fig. 8.^121^

**Fig. 9:**
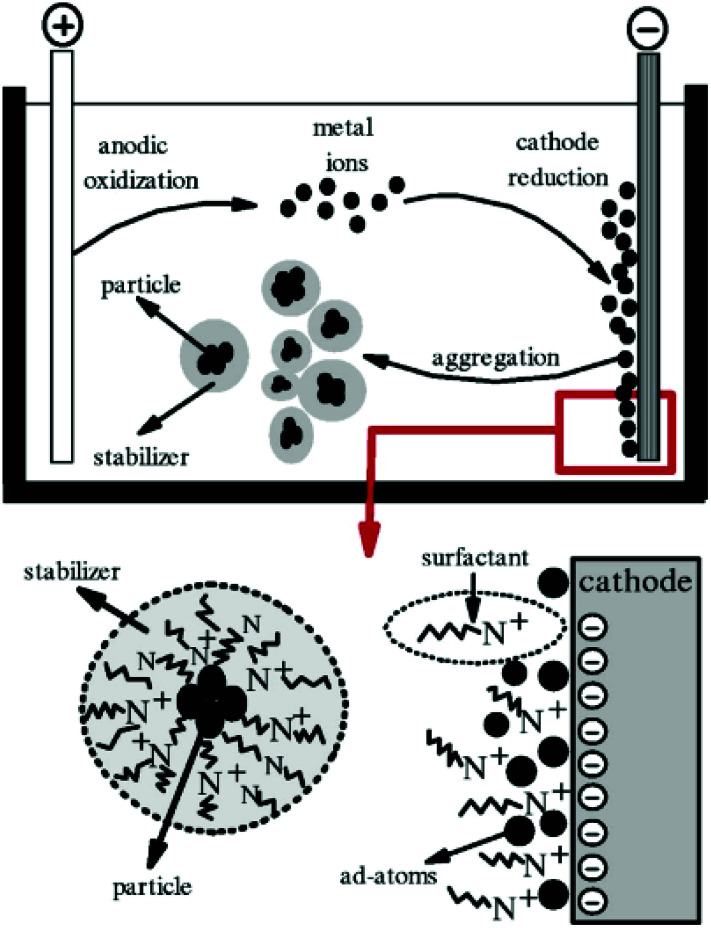
Schematic illustration of a two-electrode system for the electrochemical nanoparticle synthesis method.^191^ This figure has been adapted/reproduced from ref. 191 with permission from IOP Publishing, copyright 2006.

The electrochemical method is another common method used for the synthesis of GNPs.^136^ In general, electrochemical synthesis of GNPs is carried out in a two-electrode system. Commonly, one gold electrode and one platinum electrode are used for the electrochemical preparation of GNPs. The use of surfactants (*e.g.*, CTAB) during the electrochemical process is crucial to determine the shape of the nanoparticle. Specifically, use of C_16_TAB causes the production of spherical nanoparticles, while using the TC_12_AB support leads to the production of nanorods. The introduction of a silver plate in the electrolyte solution is also effective at providing better control over the size of nanoparticles.^62^ The experimental setup used for the electrochemical synthesis of GNPs is shown in Fig. 9.

These days researchers are also exploring biological and green chemistry methods due to environmentally friendly procedures, use of safe chemicals (in comparison to chemical methods), and less expensive production.^120,137^ Certain biological species like bacteria, plants, and fungi have capabilities to reduce metal ions to form nanoparticles;^138–140^ overall, synthesis of metal nanoparticles with fungi is preferred over bacterial synthesis methods.^141^ So far, a number of fungus species, *e.g.*, *Aspergillus fumigatus*, *Cylindrocladium floridanum*, *Fusarium oxysporum*, *Sclerotium rolfsii*, *Epicoccum nigrum*, *Candida albicans*, *Aspergillus oryzae* var. viridis, *Penicillium* sp. 1–208, and *Rhizopus oryzae*, have been utilized to prepare GNPs.^141,142^ Likewise, bacteria such as *Bacillus megaterium* D01, *Bacillus subtilis* 168, *Desulfovibrio vulgaris*, *Geovibrio ferrireducens*, *Geobacillus* sp., *Lactobacillus* strains, *Plectonema boryanum* UTEX 485, *Pseudomonas aeruginosa*, *Pseudomonas fluorescens*, *Shewanella* algae strain BRY, *Rhodopseudomonas capsulata*, *Escherichia coli*, *Citrobacter freundii*, *Citrobacter koseri*, *Proteus vulgaris*, *Serratia marcescens*, *Enterobacter* spp., *Klebsiella pneumonia*, *Proteus mirabilis*, and *Klebsiella oxytoca* have also been employed for the synthesis of GNPs.^143,144^ In the case of plant extracts, coriander, mango, *Gymnocladus assamicus*, *Pogostemon benghalensis*, olive, *Rosa indica*, *Pistacia integerrima*, *Salvia officinalis*, *Lippia citriodora*, *Pelargonium graveolens*, and *Punica granatum* have been tested as reducing agents for the gold salts to prepare GNPs.^120,137^ The studies reported during 2019–20 for the synthesis of GNPs from plant extracts, bacteria, algae, and fungi are listed in Tables 2, 3, 4, and 5, respectively.

**Table 2:** Plant extracts used for the synthesis of gold nanoparticles (published in 2019 and 2020)

Plant used for gold nanoparticle biosynthesis	Shape	Mean size (nm)	References
*Lycoris aurea*	Spherical	24	145
*Corallocarbus epigaeus*	Spherical	30	146
*Commiphora wightii*	Spherical, triangular and hexagonal	20	138
*Hypericum perforatum*	Spherical	44	147
*Solanum nigrum*	Spherical	9	148
*Origanum vulgare*	Spherical	5	149
*Clinacanthus nutans*	Spherical and hexagonal	6	150
*Chenopodium formosanum*	Spherical	8	151
*Scutellaria barbata*	Spherical	35	152
*Terminalia arjuna*	Spherical	20	153
*Etlingera elatior*	Variable	31	154
*Monotheca buxifolia*	Hexagonal, triangular and spherical	14	155
*Siberian ginseng*	Spherical	Not reported	156
*Juglans regia*	Spherical	14	157
*Anthriscus sylvestris*	Spherical	18	158
*Allium noeanum*	Spherical	20	159
*Xanthium strumarium*	Pentagonal, spherical and hexagonal	25	160
*Casa blanca*	Spherical	5	161
*Terminalia coriacea*	Not reported	55	162
*Annona muricata*	Spherical	25	163
*Kalanchoe daigremontiana*	Spherical	Variable	164
*Parkinsonia florida*	Quasi-spherical	12	165
*Caudatus geisel*	Spherical	20	166
*Origanum vulgare*	Spherical and triangular	20	167
*Zingiber officinale*	Spherical	7	168
*Sansevieria roxburghiana*	Spherical, triangular, hexagonal, rod-shaped and decahedral	17	169
*Agave potatorum*	Pseudospherical	14	170

**Table 3:** List of some bacteria used for gold nanoparticle biosynthesis (published in 2019 and 2020)

Bacteria used for gold nanoparticle biosynthesis	Shape	Mean size (nm)	References
*Marinobacter algicola*	Spherical, triangular and hexagonal	74	140
*Staphylococcus aureus*	Not reported	51	171
*Bacillus cereus*	Not reported	120	171
*Salmonella enterica*	Not reported	124	171
*Shewanella oneidensis*	Spherical	15	172
*Enterococcus* sp.	Spherical	Not reported	173
*Bacillus marisflavi*	Spherical	13	174
*Citricoccus* sp.	Spherical	45	175
*Streptomyces griseus*	Hexagonal	23	176

**Table 4:** List of some algae used for gold nanoparticle biosynthesis (published in 2019 and 2020)

Algae used for gold nanoparticle biosynthesis	Shape	Mean size (nm)	References
*Gelidium pusillum*	Spherical	12	177
*Dunaliella salina*	Spherical	22	178
*Gelidiella acerosa*	Spherical	Variable	179
*Cystoseira baccata*	Spherical	8	180
*Cystoseira tamariscifolia*	Spherical	7	180
*Halymenia dilatata*	Triangular and spherical	16	181
*Ulva lactuca*	Spherical	7	182

**Table 5:** List of some fungi used for gold nanoparticle biosynthesis (published in 2019 and 2020)

Fungi used for gold nanoparticle biosynthesis	Shape	Mean size (nm)	References
*Pichia pastoris*	Spherical	100	139
*Alternaria* spp.	Variable	28	183
*Fusarium solani*	Needle and flower shaped	42	184
*Fusarium oxysporum*	Spherical, hexagonal and triangular	Variable	185
*Fusarium oxysporum*	Spherical or hexagonal	26	186
*Macrophomina phaseolina*	Spherical	15	99
*Lentinula edodes*	Triangular, hexagonal, spherical and irregular	72	187
*Lignosus rhinocerotis*	Spherical, irregular and triangular	Variable	188
*Parmelia sulcata*	Spherical	54	189
*Fusarium oxysporum*	Spherical	Variable	190

### 4.2. Stability of the colloidal nanoparticles

GNPs exhibit many important properties that are excellent for biomedical applications. However, bare GNPs are associated with a few challenges, which need to be resolved to improve the scope of application of GNPs in biomedical engineering. One of the major challenges is to enhance their colloidal stability. (Note that bare GNPs have a natural tendency to agglomerate.) Fortunately, the stability of the GNPs can be improved by a number of techniques/approaches, *e.g.*, electrostatic stabilization, steric stabilization, phosphine ligation, thiol ligation, and ligand exchange.^192^ Among them, electrostatic and steric stabilization are the two major techniques used to stabilize nanoparticles in the liquid medium.^193,194^ In the case of electrostatic stabilization, charge–charge repulsion between nearby GNPs is used to achieve stability.^192^ The charge–charge repulsion should be greater than the van der Waals attractive forces to stabilize the particles. The electrostatic stabilization in GNPs has been achieved by functionalizing them with suitable molecules, *e.g.*, citrate and polymers.^192,195^ Likewise, in the case of steric stabilization, the aggregation of particles is restricted by steric repulsion between the molecules attached on the GNP surface.^192^ The commonly used molecules to achieve steric functionalization in GNPs are organic surfactant and polymers.^192,196^

The stability and forces between GNPs can be explained by the Lennard-Jones potential and van der Waals energy of combination.^118,197^ The Lennard-Jones potential is used to define the interaction of two molecules or atoms expressed as 
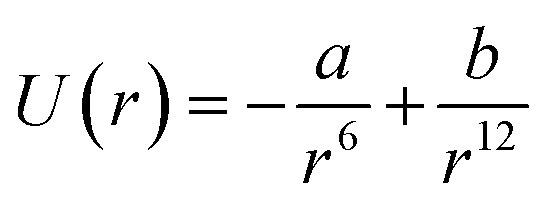
, where ‘*a*’ and ‘*b*’ are the constants related to attractive and repulsive interactions.^197^ The van der Waals energy of a combination of two particles of radius *R*, electron charge density *σ*_1_ and *σ*_2_, and inter-particle distance *r* is approximated as
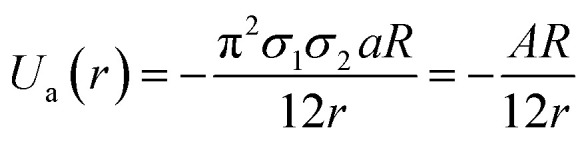
where *A* is called Hamaker's constant.^118,197^

The potential of the electric double layer (EDL) is crucial in determining the stability of GNPs.^118^ Generally, the EDL is created on the surface of colloidal particles due to interaction between the surface charges of the nanoparticles and solution ions. The EDL is comprised of a thin layer, called the stern layer (a fixed layer due to strongly bound counter ions) and a diffused layer (weakly associated). The thickness of the EDL is referred to as Debye length denoted by *λ*. In the EDL, there exists an interfacial layer (slipping plane) between the fixed ionic layer (particle + fixed layer can be treated as a single object). The measure of the potential at this layer is called zeta (*ζ*) potential.^118,197^ Likewise, DLVO theory was used to explain the stability of colloidal nanoparticles.^198,199^ The theory considers attractive van der Waals and repulsive electrostatic EDL interactions. The total interaction energy/potential is given by the following equation:*U*_total_(*r*) = *U*_a_(*r*) + *U*_r_(*r*).

Repulsive forces and hence repulsive energy usually depend on the concentration of ions, while attractive energy does not depend on the ion concentration. The steric stabilization in GNPs can be achieved by the use of high molecular weight polymers and surfactants.^200^ These molecules are chemisorbed on the surface of colloidal nanoparticles and make them stable. Overall, the stable GNPs have less free energy and high repulsive interactions.^200^ The total interaction energy of a particle can be estimated using the following equation:*U*_total_(*r*) = *U*_a_(*r*) + *U*_r_(*r*) + *U*_steric_(*r*).

## 5. Functionalization of GNPs

Functionalization of GNPs is crucial in improving the applicability of GNPs for biomedical applications. GNPs can be effectively modified with diverse chemicals and bio-molecules.^201^ GNPs are inert to several chemical reactions (*e.g.*, oxidation); nonetheless they can strongly interact with sulphur containing compounds such as thiols, disulphides, and amino acids.^12^ Interestingly, the capping of GNPs with sulfur containing compounds is beneficial for the formation of their self-assembled monolayers. Specifically, alkane-thiols are used to modify the surface chemistry of GNPs. For example, 3-mercaptopropionic acid was used to generate a free –COOH group on the surface of GNPs.^202^ The –COOH functionalized GNPs can easily bind to biomolecules, *e.g.*, antibodies, *via* carbamide bonds. Likewise, modification of GNPs with tri-*n*-octylphosphine oxide (TOPO), oleylamine, octadecylamine, triphenyl phosphine, and dodecylamine has been reported to increase the stability of GNPs in solution. Similarly, polymers such as polyacrylic acid (PAA), polyvinyl pyrrolidone (PVP), and polyvinyl alcohol (PVA) have also been used to coat the surface of GNPs.

Certain molecules, *e.g.*, tetraethylorthosilicate (TEOS), can increase the biocompatibility of GNPs by providing a silica coating on its surface.^12,22,25^ The functionalization of GNPs makes them suitable for use in aqueous as well as physiological environments. However, it is always complicated to use GNPs in physiological and cellular environments having a high concentration of ions.^204^ The high concentration of ions usually led to the aggregation of even sterically and electrostatically stabilized GNPs. Moreover, interaction of GNPs with proteins present in the bio-system can modulate the sensing results. Excitingly, functionalization of GNPs with a few molecules (*e.g.*, polyethylene glycol (PEG)) can be used to avoid non-specific binding of GNPs with proteins.^205^ PEG modification of GNPs is generally called PEGylation. PEGylation of GNPs prevents nonspecific binding of proteins to GNPs and hence improves their efficacy in the physiological environment.^205^

**Fig. 10:**
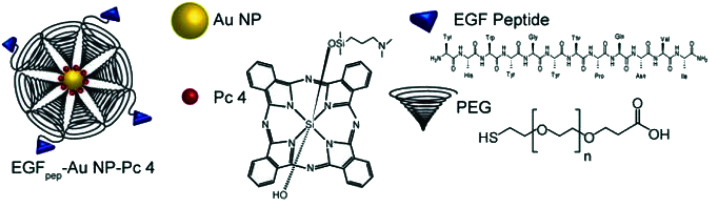
Schematic diagram of a gold functionalized material.^203^ This figure has been adapted/reproduced from ref. 203 with permission from John Wiley and Sons, copyright 2014.

The functionalized GNPs are extremely beneficial for the diagnosis and treatment of diseases (*e.g.*, cancer). In particular, the surface modification of GNPs with bio-molecules (*e.g.*, antibodies, oligonucleotides, and peptides) is found to be exceptionally helpful for cancer treatment (Fig. 10).^203,206^ Additionally, bio-functionalization of GNPs minimizes the nanoparticle–immune system interaction. The GNPs can be further modified with drug molecules such as doxorubicin, camptothecin, 5-fuorouracil, 6-mercaptopurine (anti-cancer drugs), and levodopa (anti-Parkinson's drug) for drug delivery applications.^14,40,202^

The imaging and sensing capabilities of GNPs can be explored in the form of bio-probes. GNPs have been modified with specific antibodies or antigens for effective immunosensing applications.^207^ GNPs modified with cancer specific antibodies have also been tested successfully for the targeted destruction of malignant cells without greatly affecting the healthy cells.^208^ Surface modification/coating of GNPs is also crucial for many other applications including layer by layer polyelectrolyte modification and deposition of other bio/chemical entities by a ligand exchange process.^209^ In a report, glutathione modification of GNPs was utilized in intracellular release of drugs.^22^ Moreover, the anti-bacterial and antifungal properties of GNPs are the added advantages of using GNPs in biomedical engineering applications.^210,211^ Overall, functionalization of GNPs was found to be extremely beneficial in improving stability, ability for further functionalization, sensing ability, efficiency of targeted drug delivery and imaging, and treatment capabilities.

## 6. Biomedical engineering applications of gold nanoparticles

As discussed earlier, the biomedical engineering potential of GNPs has been known to us for centuries. The extraordinary potential of GNPs is still facilitating the progressive development of the biomedical field. Some of the important biomedical engineering applications are biomedical imaging, diagnostics, nano-biosensing, nanotheranostics, nanomedicine, targeted cancer treatment, dentistry and photothermal/photodynamic therapy.

### 6.1. Bio-imaging applications

The excellent optical properties of GNPs combined with easy surface modification capability make GNPs suitable for *in vivo* and *in vitro* bio-imaging applications.^6,46,67,212,213^ Recently, the biomedical imaging applications of GNPs have drawn tremendous interest owing to their desirable size and tunable optical properties. The use of GNPs in biomedical imaging techniques such as X-ray computed tomography, photo-acoustic imaging, dark field microscopic imaging, magnetic resonance imaging, and fluorescence imaging is very popular.^21,39,68,212^ Conventionally, organic fluorophores have been used for color-based biomedical imaging.^214^ Disadvantages like the narrow excitation and broad emission spectra of organic fluorophores limited their use in imaging related applications.^215,216^ Quantum dots have also been tested widely as an alternative to fluorophores. However, quantum dots also suffer from several limitations including cytotoxicity and toxic metal ion leaching.^217^ GNPs have provided possible solutions to these issues and have many advantages over organic fluorophores and quantum dots. The presence of LSPR in GNPs is the main advantage of using GNPs. By varying the size/shape of GNPs, the LSPR wavelength can be tuned from the visible to the NIR region of the electromagnetic spectrum.^6,25^ Moreover, GNPs do not suffer from photobleaching and blinking problems.^218^

The remarkable light scattering properties of GNPs have been found to be excellent for bio-imaging applications.^41,219^ The scattering efficiency of GNPs is many times greater than that of conventional fluorophores. Interestingly, the NIR sensitive particles are efficient for dark field cell imaging.^220^ A variety of gold nanostructures, *e.g.*, gold nanoshells, nanocages, nanorods and nanostars, can be used for dark field cell imaging.^6,98,221^ Additionally, gold nanostructures are well known for SERS and have been utilized for Raman signal based imaging of biological cells.^6,22^ The quality of the formed images is distinctly comparable with that of fluorescence images.

GNPs have been proven to be a better choice for *in vivo* and *in vitro* imaging due to low toxicity, low interaction with biological components, easy synthesis, easy surface modification, and easy LSPR tunability.^222,223^ Based on the unique photo-physical properties of GNPs, many researchers have developed numerous new methodologies (*e.g.*, optical coherence tomography) for incorporation of gold nanostructures in bio-imaging.^224^ Two photon luminescence microscopy is another popular bio-imaging technique which overcomes the difficulties faced by other optical imaging methods such as auto-fluorescence of biological tissues.^225^ It can provide 3D images with high spatial resolution.

Gold nanostructures with prism, rod, and shell morphologies are suitable for photoacoustic imaging and act as exogenous contrast agents.^21,226^ Likewise, nanocages have considerably improved the photo-acoustic tomography (PAT) efficiency.^227,228^ Opto-acoustic tomography is another well-known name of PAT. PAT uses the physics principles of optical and acoustic (ultrasonic) waves to generate high contrast images.^229^ It is a rapidly growing field in bio-imaging and is readily used for clinical applications. Thermodynamics of bio-structures changes due to absorption of incident optical energy, which in turn changes the pressure (mechanical) and produces acoustic waves. The acoustic waves are detected by ultrasonic sensors and produce images. PAT has applications in blood flow monitoring, temperature monitoring, oncology, gastroenterology, neurology, cancer treatment and many more.^230^ PAT is based on the photo-acoustic effect discovered by Alexander Graham Bell and it provides a 3D cross-sectional image of biological structures like tissues, organelles, cells, and organs (*in vivo*).^227^ PAT can meet the challenges faced by optical imaging.^231^ (Note that optical imaging generally suffers from diffusion of light when deep biological visualization is required.) PAT can give 3D images of bio-structures to a considerable depth with micrometer level resolution. GNPs owing to their robust chemistry have been utilized in PAT as contrast enhancing agents.^232^ Different types of gold nanostructures such as gold shells, prisms, rods, stars, nanocages, and GNPs have been used as promising exogenous contrast agents in PAT.^21^

Immuno-electron microscopy is another very important technique in which electron microscopy is used to investigate and localize the presence of a specific protein at the sub-cellular level.^233^ This technique utilizes GNP labeled antibodies to sense, localize, and quantify specific or multiple antigens. The technique can be employed to investigate the surface or ultrathin sections of biological cells. Gold-based immuno-electron microscopy studies can also be performed *via* scanning electron microscopy.^233^ Moreover, it was also observed that nanogold is efficient in improving the scanning electron microscopic investigations of biological components.^234^

Interestingly, conventional microscopic techniques do not exhibit the capacity to pinpoint and quantify specific or multiple proteins with high resolution.^234^ Biomedical researchers have recently used GNPs for optical microscopic imaging.^234^ By using confocal microscopy, 3D images of biological specimens can be obtained. GNP–antibody conjugates provide a way of real time *in vivo* internalization of GNPs in cancerous cells.^235^ Likewise, GNPs have been utilized in dark field microscopic applications.^236^ Due to the excellent light scattering properties of GNPs, these structures appear brighter than the background. Reflectance microscopy is also one of the advanced microscopic techniques. GNPs are capable of increasing the reflection contrast in reflectance microscopy.^237^ Moreover, reflectance and confocal microscopy in combination give 3D images of the region of interest.^238^ GNPs provide considerable enhancement in X-ray computed tomography compared with conventional iodine based contrast enhancing agents.^68,212^ Gold has excellent X-ray attenuation properties (good X-ray absorption efficiency) and therefore can provide contrast to computed tomography images. Conventional iodine based contrast agents have short circulation time in blood, while GNPs show good circulation time. Excitingly, GNPs have been used in targeted cellular level detection of cancer using specific immunogens.^68,226,239,240^

**Fig. 11:**
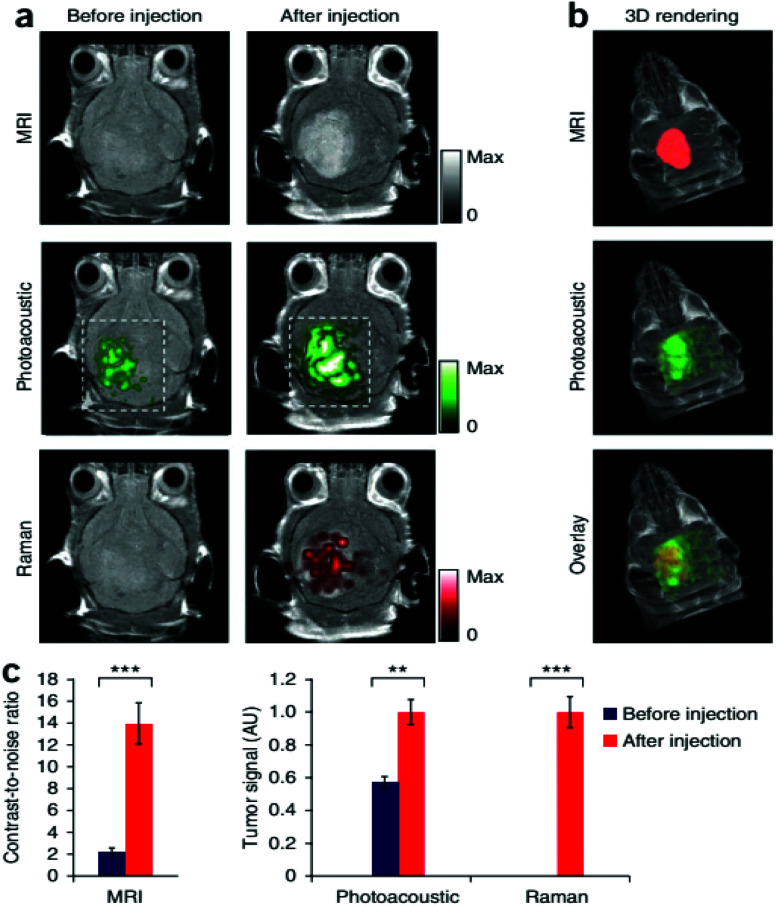
Triple-modality detection of brain tumors in living mice with MRI, photoacoustic and Raman imaging. (a) Two-dimensional axial MRI, photoacoustic and Raman images. The post-injection images of all three modalities showed clear tumor visualization (dashed boxes outline the imaged area). (b) A three-dimensional (3D) rendering of magnetic resonance images with the tumor segmented (red; top), an overlay of the three-dimensional photoacoustic images (green) over the MRI (middle) and an overlay of MRI, the segmented tumor and the photoacoustic images (bottom) showing good localization of the photoacoustic signal with the tumor. (c) Quantification of the signals in the tumor showing a significant increase in the MRI, photoacoustic and Raman signals after as compared to before the injection. *n* = 4 mice.^244^ There are some other bio-imaging methods that use GNPs like the photoluminescence method, dark field optical microscopy, Raman confocal microscopy, positron emission tomography, single positron emission computed tomography and Cerenkov luminescence imaging. All these microscopic techniques have incorporated GNPs in order to have better efficiency.^244^ This figure has been adapted/reproduced from ref. 244 with permission from Springer Nature, copyright 2012.

Magnetic resonance imaging (MRI) is another noninvasive popular modern bio-imaging technique, which gives 3D anatomical details with good resolution. The principle of MRI is related to nuclear magnetic resonance (NMR). Old MRI machines suffered from long scanning duration. The first ever MRI image was scanned in ∼5 h. Development in this field has considerably reduced the scanning time. Modern MRI machines scan the whole body within a few minutes. The performance of MRI is heavily dependent on longitudinal relaxation time (*T*_1_) and transverse relaxation time (*T*_2_).^241^ The values of *T*_1_ and *T*_2_ depend upon the type of biological material under consideration. Gadolinium based contrast enhancement agents have been widely used to obtain MRI images.^12,242^ The hybrids of gold with gadolinium chelates have also been reported for improved MRI performance.^243^ Magnetic nanocores (*e.g.*, iron, cobalt and nickel) coated with gold shells have been successfully used to enhance MRI contrast. Gold, when interacting with magnetic nanoparticles, produces magneto-plasmonic properties. These magneto-plasmonic structures have been utilized in MRI contrast enhancement as well as for optical bio-imaging. MRI–photoacoustic–Raman imaging has also been devised to have better sensitivity and to get more information in a single scan.^6,244^Fig. 11 shows the triple-modality detection of brain tumors in living mice with MRI, photoacoustic and Raman imaging.

### 6.2. Bio-sensing application

**Fig. 12:**
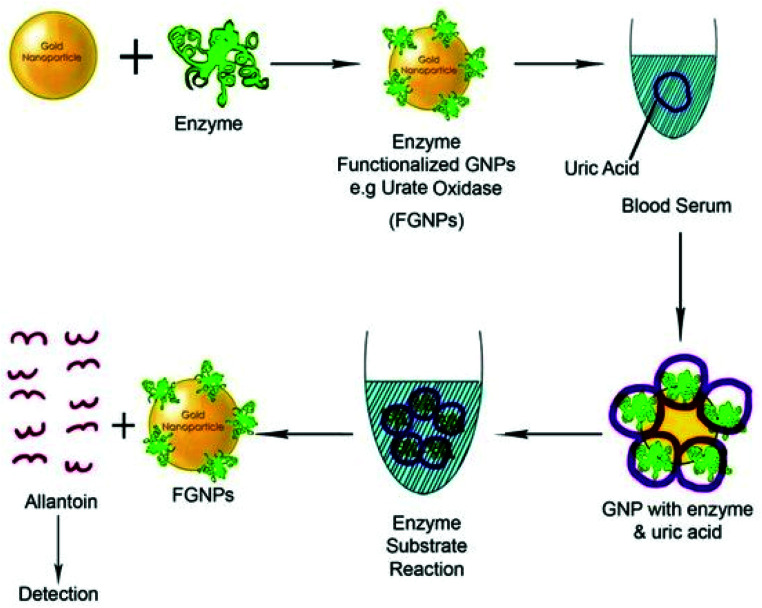
Schematic diagram of a functionalized gold biosensor to detect serum protein.^254^ This figure has been adapted/reproduced from ref. 254.

Polymer chain reaction (PCR) and enzyme-linked immunosorbent assay (ELISA) are the two most popular bio-molecular sensing methods.^245,246^ However, these methods are expensive and complicated. The use of GNP-based sensing techniques could be a game changer in the biosensing applications. So far, GNPs have also been used for efficient colorimetric, electrochemical, enzymatic, fluorescence resonance energy transfer (FRET), surface enhanced Raman scattering, and optical sensing applications.^247–249^ GNPs have been examined for the testing of choline and uric acid.^250,251^ Recently, GNPs have also been used for cardiac disease sensing, kidney malfunctioning, DNA/RNA detection, and immune-sensing (Fig. 12).^252–254^ In a study, single stranded DNA was conjugated to GNPs for the determination of the complementary DNA strand.^255^ Upon hybridization between DNA strands, the red color solution of GNPs was changed to violet/blue. The technique is sensitive enough to detect the mismatch of DNA strands. In similar fashion, effective immuno-sensing was also performed using gold nanoparticles.^256,257^ The formation of an immune complex caused aggregation of the nanoparticles, which was used to determine analyte levels through UV-vis spectroscopy.

GNPs, when in close proximity to the fluorophore, cause quenching of the fluorophore or increase its fluorescence intensity.^258^ The Raman signal is also enhanced in the presence of GNPs, which increases the sensitivity of Raman based bio-sensing.^259^ Surface plasmon resonance-based bio-engineered devices have been proven very effective in many important biological studies.^260^ GNPs due to LSPR have high electromagnetic field on their surface, which significantly increases the Raman scattered signals of bio-molecules when GNPs are present in the close proximity of bio-molecules.^261^ Hence the use of GNPs increases the Raman based sensing of bio-molecules even to very low molar concentrations. An SPR sensing device has been proven to be an excellent biomedically engineered device. The SPR device uses prisms or diffraction gratings. Real time bio-molecular interactions such as antibody–antigen interactions were effectively studied using the SPR device.^262^ Recently, graphene layers have been utilized to enhance SPR sensitivity.^6^ Thin gold nanostructures with repeated nano-scale holes (arrays) are found to be more sensitive in generating SPR and SERS signals.^262^

In the case of GNP-based colorimetric sensing, the position of LSPR is crucial.^263^ Complementary moieties, like antibodies and oligonucleotides, can be tethered to GNPs and on interaction/hybridization with the required bio-molecule cause aggregation and can be easily detected by measuring the change in LSPR characteristics.^263^ Note that the LSPR band of GNPs is broadened due to aggregation. The sensitivity and specificity of this type of sensor are very high and have great importance in early diagnostic applications. Likewise, El-Sayed *et al.* used GNPs for diagnostic applications of cancer cells.^81^ They observed that anti-epidermal growth factor receptor (anti-EGFR) monoclonal antibody grafted GNPs specifically bind to cancerous cells with greater affinity than non-cancerous cells without any aggregation.^264^ SPR signals of anti-EGFR–GNPs appeared at 536 nm, when attached to cancerous cells. The interaction between anti-EGFR–GNPs and EGFR led to the shifting of the SPR peak by 9 nm.

GNPs can provide contrast in confocal laser scanning optical microscopy, optical resonance spectroscopy, multi-photon spectroscopy, and third harmonic microscopy.^68,234,237^ Gold nanorods are better materials for *in vivo* imaging as they display two surface plasmon resonance bands (longitudinal and transverse SPR absorption bands).^227,265^ Leuvering *et al.* introduced a new term, “sol particle immunoassay” (SPIA).^266^ This technique is based on the change in optical and absorption characteristics of colloidal GNPs upon adsorption of bio-molecules onto their surfaces and on attractive interactions with target molecules. An advanced and simple version of this technique is used for detecting pregnancy in women by the presence of gonadotropin in urine.^267^ Likewise, hepatitis B detection was also achieved by an antibody–antigen immunoassay through using gold nanorods.^268^

### 6.3. Therapeutic applications of GNPs

**Fig. 13:**
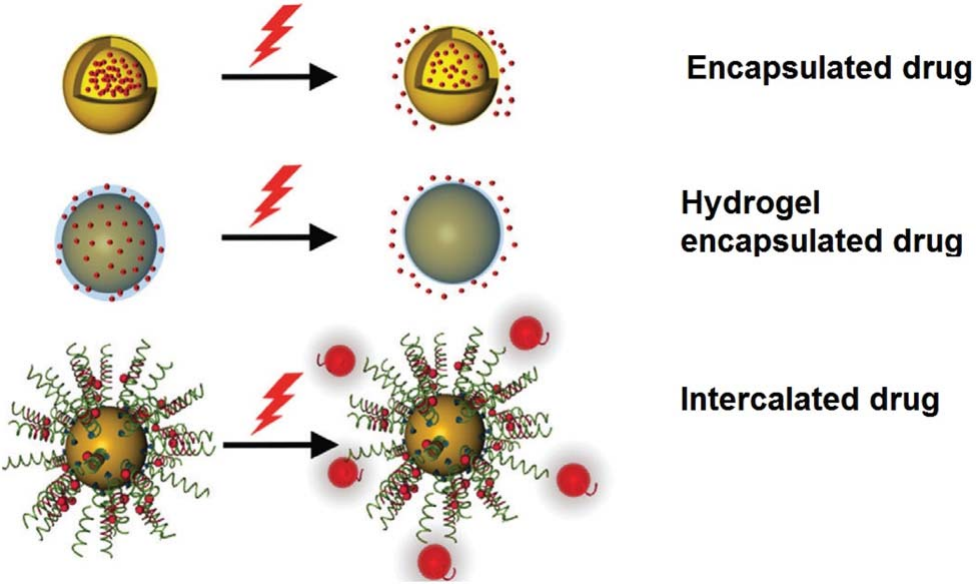
Schematic diagram showing the therapeutic application of AuNPs using light.^275^ This figure has been adapted/reproduced from ref. 275 with permission from John Wiley and Sons, copyright 2017.

The potable nano-gold has tremendous application potential to cure many diseases like rheumatic disease.^100^ From ancient times, *Swarna bhasma* has been used in India as an effective medicine to cure diseases like diabetes, rheumatoid arthritis, tuberculosis, anemia, muscular dystrophy, and many others.^269^*Swarna bhasma* can be defined as powdered gold metal with nanometer scale crystals. It is mixed with honey and milk and then orally given to patients.^269^ Interestingly, prolonged use of *bhasma* can eradicate many diseases and improve immunity. Likewise, Paracelsus has mentioned the biomedical applications of colloidal gold.^270^ He prepared aurum potabile (potable or drinkable gold) by reduction of gold salt with plant extracts in oil/alcohol.^271^ Colloidal or potable gold was used for therapeutics of *syphilis* and mental disorders.^272^ Similarly, use of gold in dental restoration has a very long history and is still used in modern dentistry.^66^ Moreover, GNPs can carry different drug molecules and bio-molecules like nucleic acids and proteins (Fig. 13).^273–277^ These nanoparticles can act in nontoxic drug and gene delivery applications.^278^

The loading (*e.g.*, adsorption and covalent bond-based loading) and unloading (*e.g.*, photo-induced unloading) of drugs can be triggered and controlled efficiently using GNP cores.^279^ GNPs are considered superior to their counterparts (*e.g.*, silver nanoparticles) for drug delivery applications due to their high stability in solution form and low cytotoxicity.^6^ Moreover, GNPs exhibit a high surface to volume ratio, hydrophilic nature, high drug or bio-entity loading capacity, and stability in physiological media, which are crucial for drug delivery applications.^280^ A number of strategies have been tested on GNPs to meet the above-mentioned features.^6^ For example, modifications of GNPs with PEG or other suitable chemicals are beneficial in lowering the toxicity and increasing the stability of GNPs.^273,281–283^ Moreover, PEG modification is also known to increase functionality on the GNP surface. Likewise, suitable surface modification of GNPs enhances their scope in biomedical engineering applications. Interestingly, it is suspected that positively charged nanoparticles can easily participate in endocytosis and phagocytosis processes.^284^ Such surface modifications in GNPs can be easily achieved by their surface modification with positively charged functional groups.^284^

In the last few decades, drug targeted drug and gene delivery has drawn immense interest.^205,285,286^ Various nano-scale carriers such as dendrimers, nanotubes, polymer structures, liposomes, nanorods, and nanoparticles (metal and semiconducting) have been tried for the delivery of drugs and genes.^11,22^ Among them, GNPs have been proven to be excellent candidates for drug and gene delivery applications due to their facile attachment, less or no toxicity, high drug/gene loading, abundance of attachment sites, and easily trackable properties.^278,287,288^ The release of the cargo in GNPs can be triggered by the internal environment (by glutathione or pH) or by external photo-induced stimulation.^69^ GNPs exhibit a large surface to volume ratio and a good amount of drugs, vectors, and genes can be immobilized on the surface of GNPs and their guided delivery to desired locations can be accomplished.^254^ In a study, the tumor necrosis factor was tethered to GNPs for antitumor activity.^45^ The attachment of PEG in addition to the tumor necrosis factor on GNPs is effective at further increasing the efficacy of the drug conjugate.^45^

The drug loading and release in GNPs can also be controlled using a laser.^45^ Khlebtsov *et al.* reported explosive destruction of nanogold shells using laser pulses which may be extremely useful in thermal killing of cancerous shells.^289^ Likewise, UV light is also useful in controlling the drug release from GNPs. For instance, 5-fluorouracil (anti-cancer drug) was attached to GNPs using a photo-responsive cross linker and the drug release was controlled by UV irradiation.^96^ Gold nanostructure-based materials are also excellent in magnetic field guided drug delivery. For example, gold coated magnetic nanoparticles (Au@MNPs) were employed successfully for magnetic field guided drug delivery applications.^290^ This material was reported to decrease the viability of cancer cells to a great extent.^290^ Likewise, this composite structure was also used to load and guide anti-rheumatic drugs (*e.g.*, methotrexate).^290^ Likewise, several other advancements have been achieved for GNPs in the field of drug delivery. Recently, in addition to drug molecules, the attachment of peptides and polysaccharides was found to be excellent for the targeted and efficient delivery of drugs to the desired sites.^291–294^

GNPs are also examined for the cellular delivery of DNA and small interfering RNA (siRNA).^209,295^ In the case of nucleic acid conjugation, the electrostatic interactions between the nucleic acid and GNPs have been explored most.^296^ Furthermore, the delivery of nucleic acids *via* GNPs can be improved by modification of GNPs with charge-reversal polymers; the charge reversal of such polymers depends upon the pH value of the surrounding environment.^209^ Moreover, permeation and retention both are improved in the case of GNPs.

Interestingly, excellent photon absorption properties have also been utilized by researchers for local thermal killing of cancerous cells.^96^ GNPs efficiently transduce photonic energy to local heating and raising the local temperature more than 44 °C, which kills the cancerous cells.^96^ The robust chemistry of GNPs allows the attachment of antibodies and vectors to their surface for specific targeting of cancerous cells and preservation of healthy/non-cancerous cells.^281^ Spherical GNPs are not able to show absorbance at NIR wavelengths.^22^ Nonetheless, the NIR absorption properties of gold nanorods, nanoshells, and nanocages can be employed for therapeutic applications.^22^ Moreover, modified GNPs are also reported to absorb in the NIR region, which is suitable for better tissue penetration.

In another GNP-based cancer therapy, an anti-mucin 7–GNP conjugate was used to destroy urothelial cancer cells by targeted photothermal therapy without affecting the healthy cells.^219^ Photons interacting with GNPs can cause a local heating effect, which can kill the cancer cells/tumors. GNPs were reported to treat human oral squamous cell carcinoma.^297^ The high photon absorption cross-section of GNPs is extremely useful for efficient photothermal therapy.^45^ Moreover, the photothermal efficacy of GNPs can be modulated/controlled by varying their sizes and shapes.

In another study, thin coating of gold on magnetic nanoparticles was found to increase thermal killing efficacy of MNPs for cancerous cells.^290^ Note that MNPs are well known for hyperthermic applications. In hyperthermia, MNPs are placed in an oscillating magnetic field to increase the temperature.

Photosensitizer-based killing of cells is also effective for cancer treatment.^96,298^ In general, photosensitizers absorb energy from an external photon energy source, which lead to the production of reactive oxygen species (ROS) and singlet oxygen (^1^O_2_). The produced ROS cause the destruction of the cancerous cells.^96,298^ After conjugation of the photosensitizer with GNPs, the production of ROS increases due to the optically sensitive properties of GNPs.^299^ To show the effectiveness of the photosensitizer and GNP conjugate, phthalocyanine/GNP hybrids were prepared and tested on mice to cure tumors.^281^ GNP based cancer treatment is very promising and believed to replace the conventional harmful methods, *e.g.*, chemotherapy and radiation therapy.

Theranostics is a new advanced biomedical engineering field that combines both therapeutics and diagnostics through a single system at the molecular level.^219^ GNPs have been proven as perfect candidates for theranostic applications as these can perform both functions quite effectively owing to their unique photo-physical properties.^32,40,300^ The most common cancer treatments (*e.g.*, chemotherapy, surgery, and radiation) exhibit many adverse side effects.^301,302^ In general, early and effective diagnosis is crucial for effective cancer treatment. Based on the imaging and nano-sensing capabilities of GNPs, site specific therapeutics (photo-induced therapy or targeted delivery of cancer treating drugs) can be performed. In a study, theranostics of urothelial cancer cells was performed using anti-mucin 7 antibody tethered GNPs.^290^ The developed GNP–anti-mucin 7 conjugate bio-probe targets malignant tumor cells. The hybrid was exposed to laser light to perform photothermal treatment of the cancerous cells. The effect of laser exposure time and laser energy was carefully investigated.^290^ Nanotheranostics allows clinicians to monitor (real time) drug molecule delivery, bio-distribution in the tumor area and treatment efficacy. So far, nanotheranostics has been applied for the treatment of diverse diseases, *e.g.*, cancer, cardiac disease, and rheumatoid arthritis.^303–306^ In particular, GNPs are used to enhance the permeability (up to a desirable level) and retention effect.^307^ In theranostics applications, nanoparticles of various sizes and shapes have been used. Examples include nano-shells, nano-spheres, and nano-stars.^308^ Recently, gold bellflower (GBF) nanoparticles have been used for theranostic applications.^40^ These nanostructures showed negligible cytotoxicity and high photon absorption capability.

Tissue engineering and regenerative medicine (TERM) is another important and rapidly growing biomedical engineering field.^309^ Tissue engineering involves the growth of functional bio-constructs on 3D scaffolds.^310–312^ On the other hand, TERM is used to regenerate and repair/heal/replace the damaged tissues or organs.^309^ TERM serves as an effective substitute to clinical organ transplantation. In particular, GNPs improve the TERM process by altering the extracellular matrix used for scaffolds.^222^ GNPs also improve cell differentiation and the intercellular active agent's delivery.^222,313^ Excitingly, GNPs are also helpful in the monitoring of intercellular processes.^314^ In a report, radioactive GNPs were prepared from gold salts containing radioactive gold isotopes.^33^ The radioactive gold, *i.e.*, ^198^Au, can be prepared by neutron bombardment on thin foil of gold. Such produced radioactive GNPs can replace conventional radioactive therapeutic agents.^315,316^ Radioactive GNPs are efficient in bio-imaging as well as cancer treatment. Interestingly, radioactive GNPs can be further modified with targeting moieties.^33^

Additionally, gold has also been used in commercialized stents.^317,318^ Many medical practitioners prefer to use gold-based stents because they can easily be visualized in X-ray imaging. Likewise, gold has been used in precision implants owing to its bio-friendly properties.^319,320^ Nowadays, gold based commercialized pregnancy kits are in trend.^321^ Overall, gold-based nanostructures demonstrated excellent potential to change the direction of the biomedical field. It can be deduced from this article that soon gold nanostructures will be an integral part of diverse biomedical fields.

## 7. Conclusion and future scope

GNPs have been used since ancient times for many applications ranging from imparting color to glasses to treatment of complicated mental disorders. Colloidal gold has a long history of being used in therapeutic applications in the form of a potable drug. Potable nano-gold has been reported to cure rheumatic diseases and mental disorders, and have been used in dental restoration. Moreover, they have also been found to improve immunity.

Optical properties of GNPs are highly sensitive to the particle shapes and sizes. LSPR is a very interesting and highly sensitive property of GNPs. At the LSPR wavelength, the GNPs exhibit strong scattering and absorption of electromagnetic radiation making them potent for biomedical applications. SPR based devices have been widely used in studying bio-molecular interactions. GNPs have also significantly improved the quality of modern bio-imaging techniques like PAT, PET, X-ray CT, SERS, and MRI. Spherical GNPs, nanostars, nanorods, and nanocages have been effectively used in biomedical engineering applications. Different types of functionalization techniques have been reported to improve the compatibility of GNPs in aqueous and physiological environments for use as bio-probes and in bio-imaging. GNPs have been used in advanced biomedical applications such as tissue engineering, regenerative medicine, and targeted drug delivery. GNPs have also been used in Ayurveda in the form of *bhasma*. GNP based targeted and photoinduced drug release has been employed in biomedical engineering applications. The photothermal properties of GNPs have been extensively used in theranostic applications. GNPs can be utilized as an alternative to chemotherapy and therefore the complications of chemotherapy can be avoided. Numerous research reports are available in the literature regarding biomedical applications of gold nanoparticles but still there are lots of research gaps and many new possibilities. The major challenges affecting the widespread use of GNPs in the biomedical field are as follows:

(1) In almost all the cases, a broad size range of GNPs has been used for specific applications. This size range of GNPs should be more confined. A broad size range may cause a decrease in the signal to noise ratio. The development of an efficient synthesis method for GNPs could be useful in attaining narrow size range GNPs.

(2) GNPs are efficient enough to replace the commonly used organic fluorophores. However, the larger size of GNPs (in comparison to that of organic fluorophores) can limit their use in some special cases. Further reduction in the particle size of GNPs could be helpful in enhancing their application prospects in broader biomedical areas.

(3) The functionalized GNPs are a boon for the biomedical field. Nonetheless, exact quantification of functional groups on GNPs is still a challenging task. The accurate quantification of functional groups on the GNP surface will be highly beneficial in deciding the loading of exact numbers of drug/imaging agent molecules.

(4) The higher cost and limited availability of GNPs in the market are other major issues, which restricted their extended applicability in the biomedical field. However, both of these issues could be managed by large scale production of GNPs.

Overall, suitable control over nanoscale size, shape, functionalization, and cost can make GNPs an excellent candidate for advanced biomedical applications.

## Conflicts of interest

There are no conflicts to declare.

## Supplementary Material
